# Cell-to-Cell Signaling Influences the Fate of Prostate Cancer Stem Cells and Their Potential to Generate More Aggressive Tumors

**DOI:** 10.1371/journal.pone.0031467

**Published:** 2012-02-06

**Authors:** Luisa Salvatori, Francesca Caporuscio, Alessandra Verdina, Giuseppe Starace, Stefania Crispi, Maria Rita Nicotra, Andrea Russo, Raffaele Adolfo Calogero, Emanuela Morgante, Pier Giorgio Natali, Matteo Antonio Russo, Elisa Petrangeli

**Affiliations:** 1 Institute of Molecular Biology and Pathology, CNR, Rome, Italy; 2 Department of Therapeutic Program Development, CRS, Regina Elena Cancer Institute, Rome, Italy; 3 Department of Experimental Medicine, Sapienza University of Rome, Rome, Italy; 4 Gene Expression Core - Human Molecular Genetics Laboratory, Institute of Genetics and Biophysics, CNR, Naples, Italy; 5 Department of Surgical Pathology, Regina Elena Cancer Institute, Rome, Italy; 6 Bioinformatics and Genomics Unit, Molecular Biotechnology Center, Turin, Italy; 7 CINBO Laboratories, University of Chieti, Chieti, Italy; 8 Department of Cellular and Molecular Pathology, IRCCS San Raffaele Pisana, Rome, Italy; Consiglio Nazionale delle Ricerche (CNR), Italy

## Abstract

An increasing number of malignancies has been shown to be initiated and propelled by small subpopulations of cancer stem cells (CSC). However, whether tumor aggressiveness is driven by CSC and by what extent this property may be relevant within the tumor mass is still unsettled. To address this issue, we isolated a rare tumor cell population on the basis of its CD44^+^CD24^−^ phenotype from the human androgen-independent prostate carcinoma cell line DU145 and established its CSC properties. The behavior of selected CSC was investigated with respect to the bulk DU145 cells. The injection of CSC in nude mice generated highly vascularized tumors infiltrating the adjacent tissues, showing high density of neuroendocrine cells and expressing low levels of E-cadherin and β-catenin as well as high levels of vimentin. On the contrary, when a comparable number of unsorted DU145 cells were injected the resulting tumors were less aggressive. To investigate the different features of tumors *in vivo*, the influence of differentiated tumor cells on CSC was examined *in vitro* by growing CSC in the absence or presence of conditioned medium from DU145 cells. CSC grown in permissive conditions differentiated into cell populations with features similar to those of cells held in aggressive tumors generated from CSC injection. Differently, conditioned medium induced CSC to differentiate into a cell phenotype comparable to cells of scarcely aggressive tumors originated from bulk DU145 cell injection. These findings show for the first time that CSC are able to generate differentiated cells expressing either highly or scarcely aggressive phenotype, thus influencing prostate cancer progression. The fate of CSC was determined by signals released from tumor environment. Moreover, using microarray analysis we selected some molecules which could be involved in this cell-to-cell signaling, hypothesizing their potential value for prognostic or therapeutic applications.

## Introduction

Prostatic adenocarcinoma (PCa) is a leading cause of death among men in the United States and Western Europe [1]. Because of its androgen-dependent growth, hormone ablation remains the main treatment of metastatic disease. While initially effective, this treatment is followed in a few years by tumor recurrence [2] in which an androgen-independent neuroendocrine (NE) subpopulation of cells is thought to play an important role [3], [4]. NE cells are quiescent, terminally differentiated cells characterized by dendrite-like processes extending between adjacent cells and by the expression of neuronal-like proteins such as CD56 and chromogranin A (CGA) contained in dense cytoplasmic granules [5]. Through the secretion of neuropeptides NE cells modulate the activity of normal prostate epithelium but are also capable to influence adjacent transformed epithelial cells via paracrine signals, thus stimulating tumor growth and metastatic capacity [5]–[7]. In fact, an increased NE cell population in PCa is thought to be associated with a more aggressive disease, whereas a low number of NE cells in tumor tissue have no specific prognostic meaning [6], [8], [9]. Interestingly, both NE and secretory epithelial lineage are derived from a common pluripotent prostate stem cell [10].

A further basic mechanism involved in the progression of PCa is decreased expression of E-cadherin, the main transmembrane adhesion molecule responsible for cell-to-cell interactions and tissue organization in epithelial cells [11], [12]. Through the cytoplasmic domain, it binds β-catenin, which influences cytoskeletal arrangement [13]. As a consequence, loss of E-cadherin function or expression is considered a crucial event in the disruption of cell-cell adhesion and cytoskeletal architecture and in the acquisition of an invasive phenotype in tumor cells [14]. In particular, in PCa, lower expression of E-cadherin was associated with more advanced tumor stage and grade [15], [16]. Poorly differentiated prostate tumors also showed higher expression of vimentin, a cytoskeletal component responsible for maintaining cell integrity, and high levels of vimentin correlated with the invasive capacity of prostate cancer cell lines, including DU145 [17].

Traditionally, tumors have been considered to be composed of heterogeneous cells with comparable unlimited proliferative and tumorigenic potential. However, it has recently been hypothesized that only rare cells within the tumor, named cancer stem cells (CSC), are able to proliferate extensively and are tumorigenic, whereas the majority of cells in the tumor mass show a variable degree of differentiation and undergo a limited number of divisions. Their contribution to tumor growth and metastatization is considered to be rather limited. Importantly, this model implies the need for a new therapeutic approach specifically targeted towards the CSC in the attempt to definitively eradicate the tumor [18]. However, whether tumor aggressiveness is driven by CSC and by what extent this property may be biologically relevant within the naturally occurring tumor mass is still unsettled.

As the presence of CSC in PCa [19] and prostate cancer cell lines [20] was recently demonstrated on the basis of the surface antigenic profile CD44^+^/α_2_β_1_^hi^/CD133^+^ and CD44^+^CD24^−^, respectively, in the present study we aimed to evaluate the contribution of CSC to tumor progression. We isolated CD44^+^CD24^−^ stem-like cancer cells from the androgen-independent prostate cancer cell line DU145 derived from a brain metastasis of human PCa, showed their CSC properties, and investigated their phenotype and behavior with respect to the bulk DU145 cells. Importantly, in this model of prostate cancer we observed that CSC were able to generate highly aggressive tumors in NOD/SCID mice and that this potential was limited by the presence of differentiated DU145 cells with the consequent growth of less aggressive tumors. Consistently, by growing CSC in conditioned medium from DU145 cells *in vitro*, we unveiled that diffusible factors released from differentiated tumor cells were able to restrain CSC from differentiating into cell populations showing an aggressive phenotype and with features similar to those of cells held in tumors generated in mice from the injection of CSC. Finally, functional analysis of genes found differentially expressed in CSC and DU145 cells by microarray analysis confirmed the essential role of cell-to-cell signaling molecules to determine the behavior of CSC. Further investigations performed also in other models of prostate cancer might assign prognostic or therapeutic value to this signature.

## Results

### Isolation and characterization of CSC from DU145 prostate cancer cells

**Figure 1:**
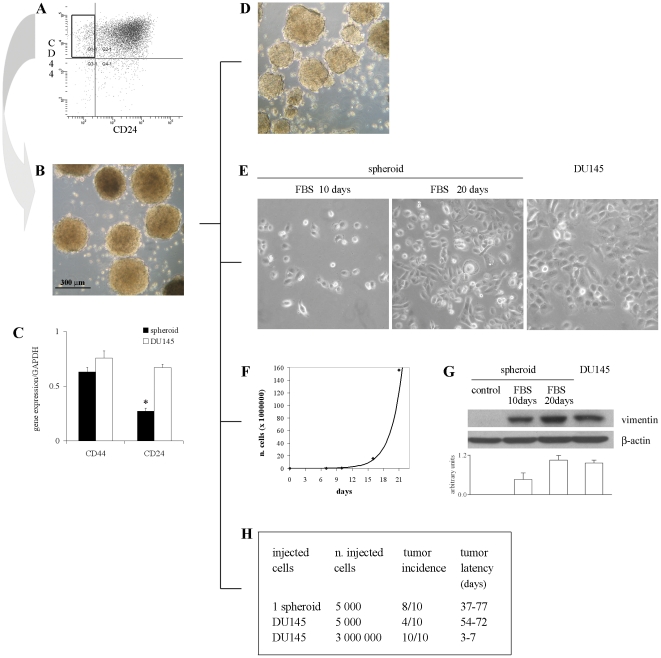
Sorting, culture and characterization of stem-like cells from DU145 cell line. (**A**) FACS analysis of CD44 and CD24 expression in DU145 cells. (**B**) Culture of isolated CD44^+^CD24^−^ cells growing in SRM as nonadherent prostaspheres (5× objective). (**C**) Q-PCR of CD44 and CD24 expression in spheroid and DU145 cells. Data are presented as mean ± SEM from 3 experiments. *, *p*<0.05 *vs* DU145 cells. (**D**) Self-renewal capacity of spheroids. Serum supplementation and the withdrawal of growth factors induce the growth of spheroid cells as adherent cells with morphology (**E**), proliferation rate (**F**) and expression of vimentin (**G**) comparable to DU145 cells. Photographs of spheroid cells were taken under a phase contrast microscope after 10 and 20 days of growth in FBS-containing medium (20×). The representative western blots show the expression of vimentin and β-actin in spheroid cells grown in FBS-supplemented medium with respect to control spheroids and DU145 cells. The histogram displays the densitometric quantification of vimentin normalized to β-actin levels. Values are expressed as mean ± SEM from 3 independent experiments. (**H**) Tumor incidence and latency after injection of 1 spheroid, 5,000 or 3×10^6^ DU145 cells in NOD/SCID mice.

DU145 cells were enriched by fluorescence-activated cell sorting (FACS) for the population expressing CD44^+^CD24^−^ [20] which represented about 10% of bulk DU145 cells (Figure 1A). We tested the stem-like properties of selected cells to indisputably describe them as prostate CSC. A very small percentage of CD44^+^CD24^−^ isolated cells was able to generate non-adherent spherical clusters, termed spheroids or prostaspheres, in serum-free medium supplemented with EGF, bFGF and insulin (Serum Replacement Medium, SRM). Spheroids grew very slowly, reaching the size of about 300 µm within 6–8 weeks (Figure 1B). As expected, spheroids were enriched for CD44^+^CD24^−^ cells, as determined by both immunofluorescence (IF) staining (data not shown) and quantitative real-time PCR (Q-PCR) analysis, with comparable results. In fact, in spheroids CD44 mRNA was expressed at levels similar to DU145 cells, whereas the expression of CD24 was significantly lower (Figure 1C).

Spheroids contained long-term self-renewing cells, as demonstrated by the ability of a fraction of cells from dissociated spheroids to generate new prostaspheres lasted over 5 passages (Figure 1D).

We observed that spheroid cells displayed differentiation capacity. In fact, when removed of growth factors and exposed to 10% FBS-containing medium, a significant fraction of spheroid cells became adherent, grew as a flat monolayer and showed a heterogeneous morphology highly similar to DU145 cells after 20 days (Figure 1E). In the early days of growth in these permissive conditions, spheroid cells showed a doubling time of about 50 h that progressively decreased up to the proliferation rate of DU145 cells, namely 30 h, starting from 15 days of culture (Figure 1F). Moreover, to confirm the appearance of the phenotype of DU145 differentiated cancer cells we analyzed the expression of the intermediate filament protein vimentin. In fact, vimentin expression has been reported to be modulated during cell maturation according to the cell lineage [21] and was found highly expressed in DU145 cell line [17]. Actually, after 20 days in FBS-supplemented medium, vimentin-negative cells derived from spheroids expressed vimentin at levels comparable to DU145 cells (Figure 1G).

Finally, we determined the tumor-producing ability of spheroid cells with respect to unsorted DU145 cells into immunodeficient mice. The injection of 1 spheroid of 300 µm in diameter, containing approximately 5,000 cells, resulted in tumors in 80% of mice, whereas the injection of 5,000 bulk DU145 cells generated tumors in 40% of animals (Figure 1H). Moreover, the tumor latency was also shorter in the spheroid-injected mice than in the mice injected with unsorted cells (37 and 54 days, respectively). The injection of 3×10^6^ DU145 cells, which were used as controls, generated tumors with the lowest latency in all the mice. Consistent with a previous report [20], all together our results indicate that CD44^+^CD24^−^ spheroid cells present key biological properties of CSC since they showed capacity for self-renewal, were able to differentiate into parental DU145 cells and were more tumorigenic than bulk DU145 cells when the same number of cells was injected in mice.

### Heterogeneous composition of spheroids

**Figure 2:**
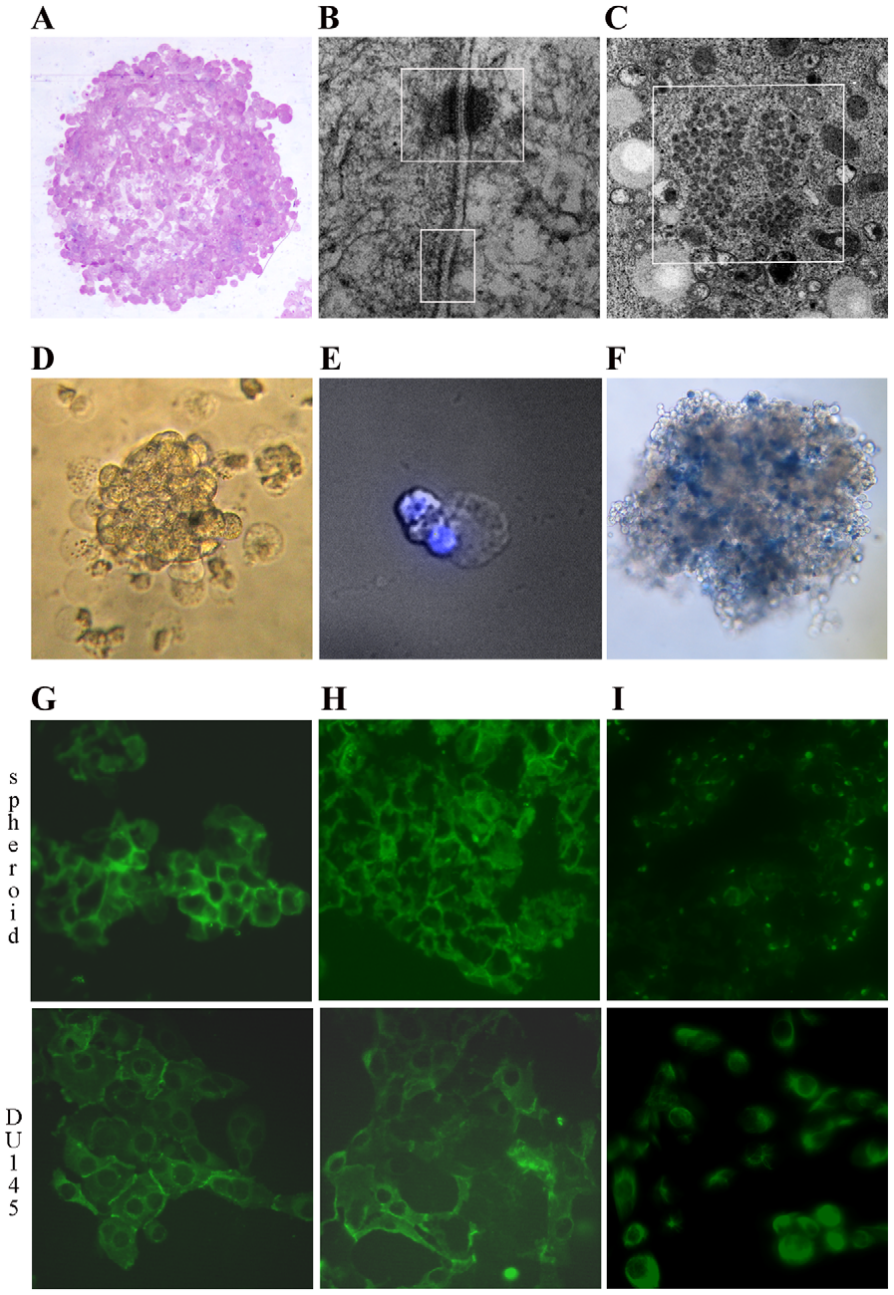
Structure and composition of spheroids. (**A**) Phase contrast evaluation of spheroid structure (10×). (**B**) TEM analysis showing desmosomes and adherent junctions (top and bottom, respectively) as well as the annulate lamellae (**C**) in spheroid cells. (**D**) Phase contrast (20×) of a fragment of spheroid surrounded by cells characterized by protruding blebs of empty cytoplasm, which present intact nuclei as shown by DAPI staining (40×, **E**). (**F**) Staining with trypan blue shows the presence of dead cells in spheroids (10×). Immunofluorescence staining of a section of spheroids and DU145 cells (original magnification ×200) showing the expression of E-cadherin (**G**), β-catenin (**H**) and vimentin (**I**).

Spheroids up to 300–400 µm in diameter showed a compact architecture (Figure 2A) due to the presence of very tight cell-to-cell contacts, as seen on Transmission Electron Microscopy (TEM). High-power magnification showed multiple junctional complexes formed by desmosomes and adherent junctions (Figure 2B). Spheroid cells also presented nuclear pore complexes organized in annulate lamellae (Figure 2C), which are considered precursors in the assembly of the nuclear envelop. They are typically present in embryonic and immature cells and disappear during differentiation [22]. However, spheroids also hold differentiated cells that, in these culture conditions selective for stem cells, showed several levels of cell degradation. In particular, characteristic cytoplasmic blebs were evident (Figure 2D), indicating that the degrading phase approached cell necrosis (Figure 2F), while nuclei appeared mostly intact (Figure 2E). These damages suggest the terminal destiny of differentiated cells.

Spheroids and DU145 cells were also evaluated for the expression of markers of stem (E-cadherin and β-catenin [23]–[25]) and differentiated cells (vimentin). Immunofluorescence staining identified E-cadherin and β-catenin expression in both cell populations, but at higher levels in spheroids (Figures 2G and 2H, respectively). Notably, in spheroid cells E-cadherin and β-catenin staining was localized on the plasma membrane, whereas in DU145 cells it was mainly cytoplasmic, indicating that differentiated tumor cells were quite independent of cell-to-cell interactions. The mature phenotype of DU145 cells was accompanied by a high expression of vimentin, which, on the contrary, was expressed only by rare cells in spheroids (Figure 2I). Therefore, we can conclude that spheroids are enriched for CSC but also held some damaged differentiated cells destined to necrosis.

### Analysis of tumors grown in mice

Comparing tumors generated in NOD/SCID mice after injection of 1 spheroid (containing approximately 5,000 total cells, considering both CSC and damaged differentiated cells) or 5,000 unsorted DU145 cells (which contain mainly differentiated cells and a very small percentage of CSC), several macroscopic differences were evident at the time of tumor excision. Tumors from spheroids grew deeply at the site of injection, invaded adjacent tissues and showed marked vascularization. Differently, tumors originating from DU145 cell injection grew superficially and appeared well delimited and mobile in relation to the surrounding tissues. Visible vessels were rare or absent (data not shown).

**Figure 3:**
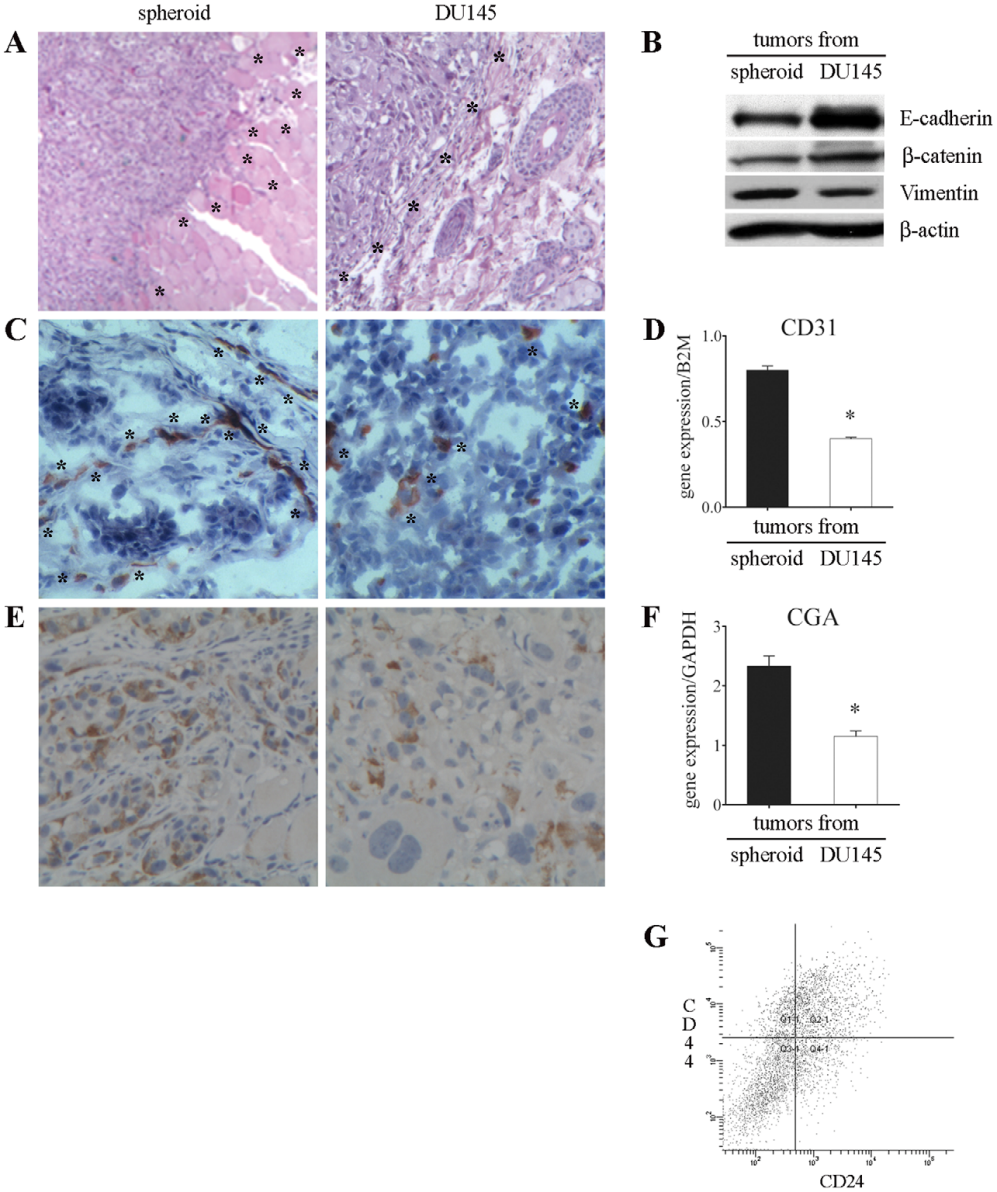
Analysis of tumors generated in NOD/SCID mice. (**A**) Histological staining with H&E of tumors grown following the injection of 1 spheroid or 5,000 DU145 cells showing highly invasive (left panel) and scantly invasive (right) phenotype (5×). Asterisks indicate the borders between tumors and the surrounding tissues. (**B**) Representative western blots showing the expression of E-cadherin, β-catenin and vimentin in tumors. (**C**) IHC staining with anti-mouse CD31 antibody highlighting higher vessel density in spheroid-derived tumors (indicated by asterisks, left panel) compared to tumors from DU145 cells (right). (**D**) Q-PCR showing mouse CD31 expression in tumors. Mouse B2M was used as endogenous control. Values are mean ± SEM from 3 experiments. *, *p*<0.05 relative to tumors generated from spheroid. (**E**) IHC with anti-CD56 antibody showing higher density of NE cells in tumors generated from spheroid than from DU145 cells (20×). (**F**) Q-PCR displaying CGA mRNA level in tumors. Results are expressed as ratios between the target gene and the endogenous control GAPDH and represent mean ± SEM from 3 independent experiments. *, *p*<0.05 with respect to tumors generated from spheroid. (**G**) A representative FACS analysis showing the presence of CD44^+^CD24^−^ cells in tumors generated from the injection of 1 spheroid in mice. Tumors originating from the injection of 5,000 DU145 cells showed a comparable CD44^+^CD24^−^ cell population (data not shown).

These differences were confirmed by histological analysis. Spheroid-derived tumors were composed of monomorphic cells with a high nucleus/cytoplasm ratio and presented a marked infiltrative growth characterized by bands invading the surrounding muscular and adipose tissues (Figure 3A, left panel). On the contrary, tumors generated from unsorted DU145 cells were composed of morphologically heterogeneous subpopulations of cells and showed an expansive growth, compressing rather than infiltrating the adjacent host tissues (Figure 3A, right panel). Interestingly, highly invasive tumors showed lower expression of E-cadherin and β-catenin and higher expression of vimentin when compared to scarcely invasive tumors (Figure 3B). Notably, in each tumor the pattern of expression of these 3 markers was opposite to that of the cells which originated the tumor itself, namely CSC or DU145 cells (Figures 2G–2H).

Tumors generated from spheroids showed also a significant higher number of blood vessels (*p*<0.0001), as monitored by staining with the endothelial cell marker CD31 (Figure 3C, left), when compared to tumors originated from DU145 cells (Figure 3C, right). These results were confirmed by the analysis of CD31 mRNA expression level (Figure 3D) and indicate active neoangiogenesis in tumors generated from spheroids. In addition, immunohistochemical (IHC) analysis showed a higher number of cells positive for CD56, a marker of NE cells, in more invasive tumors than in less invasive tumors (Figure 3E). This observation was confirmed by real-time PCR analysis of CGA, a further marker of NE cells (Figure 3F). Furthermore, FACS analysis of tumors excised and digested revealed that both types of neoplasia contained a comparable CD44^+^CD24^−^ cell population (Figure 3G). These findings provide evidence that CSC were able to generate highly invasive tumors in nude mice. However, injection of CSC with DU145 differentiated tumor cells (i.e. injection of unsorted DU145 cells) resulted in the growth of tumors of low degree of aggressiveness.

### Effect of conditioned medium from DU145 cells on the differentiation of CSC

**Figure 4:**
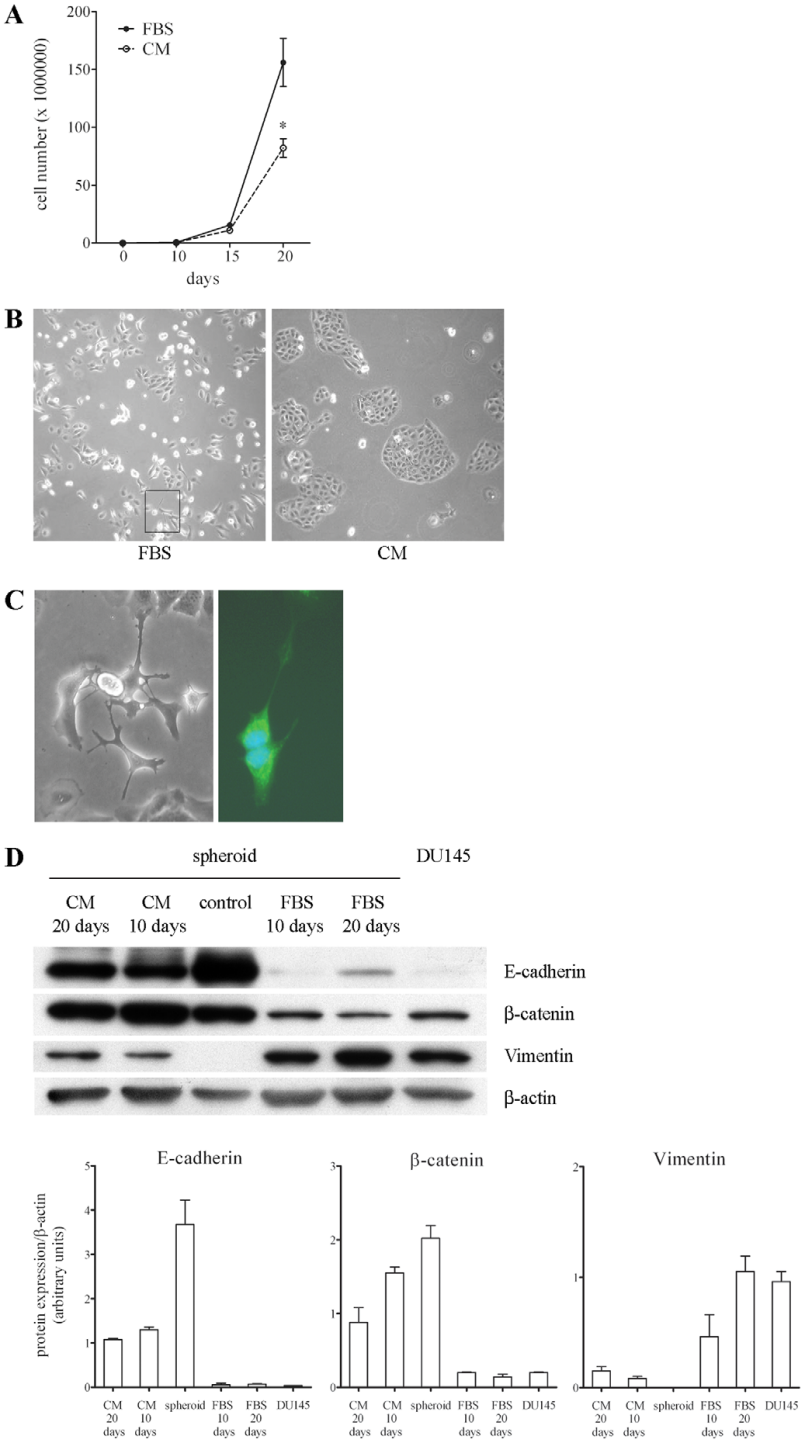
Effect of CM from DU145 cells on the phenotype of CSC. (**A**) The proliferation of CSC contained in spheroids grown in FBS-containing medium or in CM from DU145 cells was evaluated at different times of culture. Data are shown as the mean ± SEM from 3 experiments. Asterisks indicate a significant inhibitory effect of CM with respect to FBS-medium (*, *p*<0.05). (**B**) Phase contrast (10×) of cells after 20 days in FBS-containing medium or in CM showing the different pattern of cell growth. In the left panel, the rectangle highlights the presence of NE cells. (**C**) Phase contrast (left, 20×) and IF staining (right, 40×) of branched NE cells generated from CSC grown in FBS-medium. In particular, the right panel shows the expression of CD56 in the cytoplasm and in the long cell processes. (**D**) Representative western blots showing the changes in the expression of E-cadherin, β-catenin, and vimentin in spheroid CSC grown in the different culture conditions with respect to control spheroids and DU145 cells. The expression levels of the 3 proteins were determined by densitometric analysis of the respective bands and normalized to β-actin levels. Values reported in the histograms are mean ± SEM from 3 independent experiments.

In view of the above findings, we investigated the influence of DU145 differentiated cells on selected CD44^+^CD24^−^ prostate CSC *in vitro*. Dissected spheroids were grown in DMEM supplemented with FBS (to simulate the injection of spheroids in mice) or in DMEM supplemented with FBS in which DU145 cells were previously grown for 3 days (conditioned medium (CM), to simulate the injection of bulk DU145 cells in mice). In both culture conditions, CSC contained in spheroids grew in monolayer, but their proliferation was significantly reduced when they were cultured for 20 days in CM (−47%; Figure 4A). The inhibition of spheroid proliferation was further enhanced by increasing the concentration of CM (data not shown). Moreover, it appeared very evident that cells grown in FBS-containing medium were quite independent from cell-to-cell interactions as they grew mainly singly or in little groups with irregular shape, and their morphology and pattern of growth were similar to those of DU145 cells (Figure 4B, left panel). On the contrary, cells in CM were tight each other and grew mainly as roundish islands (Figure 4B, right panel). Interestingly, after 20 days and only in the plates containing FBS-supplemented medium, we observed the appearance of cells exhibiting the dendrite-like morphology typical of NE cells (Figure 4B, left panel and Figure 4C). The identity of these cells was confirmed by the evaluation of CD56 expression in the cytoplasmic granules of NE cells (Figure 4C, right panel). The analysis of the expression of markers of stem and differentiated cells highlighted that after 20 days in FBS-containing medium, cells displayed levels of E-cadherin, β-catenin and vimentin comparable to those shown by DU145 cells (Figure 4D). On the contrary, cells grown for 20 days in CM exhibited levels of expression intermediate between those shown by control CSC and DU145 cells but closer to the levels of CSC. In particular, a reduction in the expression of E-cadherin and β-catenin and a slight increase in the level of vimentin with respect to control spheroids was evident (Figure 4D). It is of interest to note that both the high expression of adhesion molecules and the low expression of vimentin indicated strong cell-to-cell interactions and reduced cell motility, respectively, which agreed well with the peculiar cell growth as isles. These results indicate that CSC cultured in permissive conditions were able to generate terminally differentiated cells, namely DU145 and NE cells. However, diffusible factors released from DU145 cells prevented the differentiation of CSC.

### Gene expression profile of CSC and DU145 cells

To investigate the cellular response involved in tumorigenesis both at the level of gene expression and at the pre-mRNA splicing level, we performed a whole genome, splicing-sensitive microarray analysis of DU145 cells and their selected CSC. RNA samples prepared from each cell population were hybridized to Human Exon 1.0 ST Arrays, which allow the definition of both transcription patterns (gene-level analysis) and alternative pre-mRNA maturation events (exon-level analysis). Gene-level expression profiling was detected by linear model statistics using an empirical Bayes method to moderate the standard errors. To evaluate transcriptional modifications, we analyzed gene expression changes in spheroid CSC *vs* DU145 cells. To identify differentially expressed genes, we applied an absolute log_2_-fold change ≥+/−1 and a P-value≤0.05 as cutoffs. The complexity of the data set was reduced by removing the non-significant probe sets (those not expressed and those not changing). In this way, more than 400 genes whose levels were significantly different in CSC and DU145 cells were selected. A complete list of the differentially expressed genes is found in Table S1.

### Analysis of selected genes

**Figure 5:**
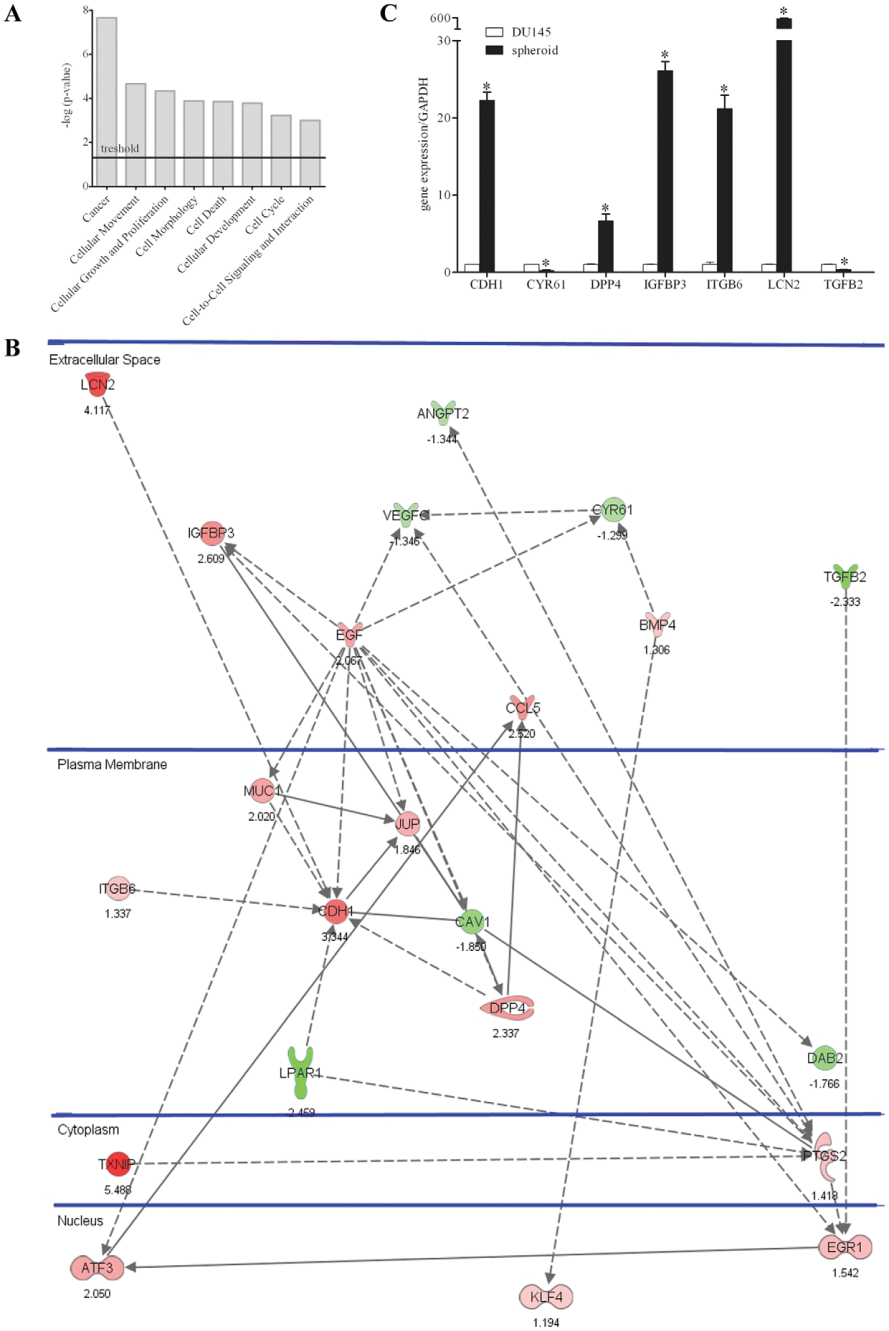
Analysis of CSC *vs* DU145 cells by microarray, IPA and Q-PCR analysis. (**A**) Selected 8 top biological functions found enriched within the set of transcripts modulated in CSC with respect to total DU145 cells by microarray analysis. (**B**) The 22 deregulated genes, as determined by IPA, are positioned in subcellular layouts, and relationships are marked by arrows: filled and dashed line arrows mark direct and indirect interactions, respectively. Genes in red showed increased expression in CSC, while genes in green were down-regulated. (**C**) Differences in gene expression determined by microarray analysis were confirmed by Q-PCR, choosing 7 representative genes among selected 22. Results are expressed as ratio between each target gene and the endogenous control GAPDH. Expression in CSC was compared to what was observed in DU145 cells to which a value equal to 1 was arbitrarily assigned. Values are mean ± SEM from 3 experiments. *, *p*<0.05 relative to DU145 cells.

The differentially expressed genes were analyzed for their molecular and cellular functions and associated pathways using the Ingenuity Pathways Analysis software (IPA 7.0, Ingenuity Systems). The IPA analysis identified more than 70 functional classes, and among them, we selected the 8 most consistent classes found enriched within the set of differentially expressed genes (i.e., cancer, cellular movement, cellular growth and proliferation, cell morphology, cell death, cellular development, cell cycle, cell-to-cell signaling and interaction; Figure 5A) that were suited to characterize CSC and DU145 cells. We combined the genes from the 8 selected functional classes, detecting 22 common genes still showing functional links. These were then separated according to their cellular localization. Interestingly, further analysis of the sub-selected genes showed that 17 out of 22 gene products were localized on the plasma membrane or were secreted in the extracellular space (Figure 5B), strengthening our above findings showing the importance of the interactions between CSC and the microenvironment. Although the selected genes encoded proteins with a wide variety of functions, 4 main groups could be identified: 1) Pro-angiogenic factors angiopoietin 2 (ANGPT2), vascular endothelial growth factor C (VEGFC) and cysteine-rich angiogenic inducer 61 (CYR61) were found down-regulated in CSC with respect to DU145 cells; 2) Genes coding for adhesion molecules E-cadherin (CDH1), integrin beta 6 (ITGB6) and junction plakoglobin (JUP) were expressed more in CSC in agreement with the parallel down-regulation of caveolae protein (CAV1); 3) Tumor suppressor genes lipocalin 2 (LCN2), dipeptidyl peptidase-4 (DPP4), insulin-like growth factor binding protein 3 (IGFBP3), thioredoxin interacting protein (TXNIP) and Kruppel-like factor 4 (KLF4) were up-regulated in CSC; 4) Diffusible factors relevant in the maintenance of stem cell niche such as epidermal growth factor (EGF), bone morphogenetic protein 4 (BMP4) and transforming growth factor beta 2 (TGFB2) were also differently expressed in CSC and DU145 cells. Noteworthy, 19 out of 22 genes that we identified were not previously described to be abnormally expressed in prostate CSC. These data confirm that interactions with the microenvironment have a crucial role in the control of the phenotype of CSC and suggest a limited number of genes whose relevance in CSC signaling will need to be investigated. Moreover, most of the 22 selected genes could be novel markers of prostate CSC.

### Validation of gene expression

To confirm the differences in gene expression found by microarray analysis, Q-PCR was performed for 7 differentially expressed genes (CDH1, CYR61, DPP4, IGFBP3, ITGB6, LCN2, TGFB2). In all cases, the differences in the mRNA levels were confirmed (Figure 5C).

### Role of the pathway of E-cadherin in the differentiation of CSC

**Figure 6:**
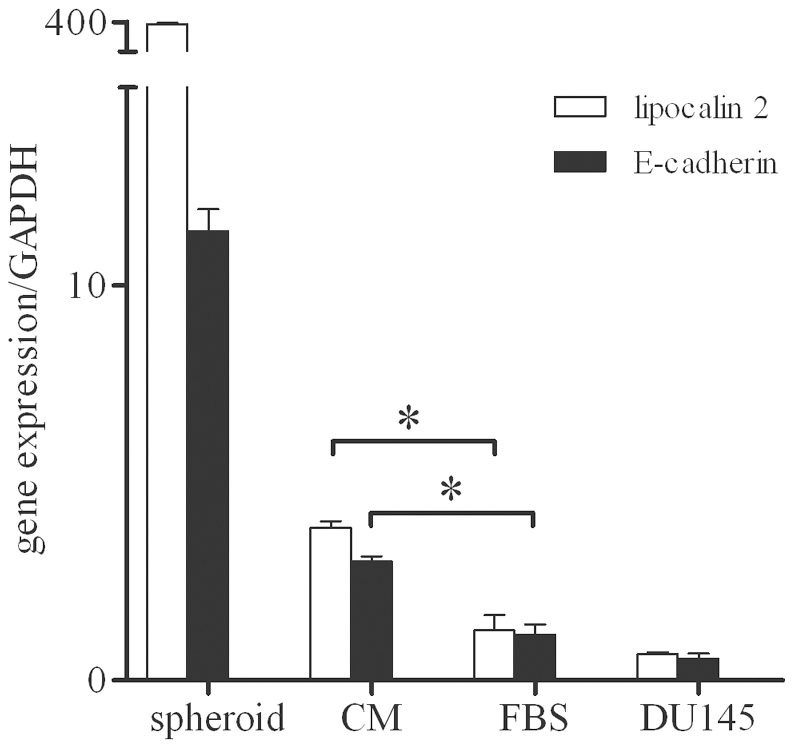
Influence of culture conditions on the expression of lipocalin-2 and E-cadherin in CSC. Q-PCR showing changes in mRNA levels for lipocalin-2 and E-cadherin in DU145 cells, CSC contained in control spheroids, and CSC grown for 20 days in FBS-medium or CM from DU145 cells. Data represent mean ± SEM from 3 individual experiments. Asterisks indicate a significant difference (*, *p*<0.05).

Q-PCR analysis showed that mRNA expression of lipocalin 2 and E-cadherin followed a comparable trend in CSC, DU145 cells and in CSC grown for 20 days in FBS-containing medium or in CM. In particular, mRNA levels of both genes were very high in CSC contained in spheroids, were reduced in CSC grown in CM and were significantly lower and comparable in CSC grown in FBS-medium and in DU145 cells (Figure 6). This trend was in agreement with the modulation of β-catenin expression previously observed in the same culture conditions (Figure 4D). Collectively, these results indicate that the pathway of E-cadherin was modulated during CSC differentiation.

## Discussion

### Tumorigenicity of prostate CSC

CD44^+^CD24^−^ cells isolated from DU145 prostate carcinoma cell line using cell surface markers were able to grow as non-adherent spheroids in serum-free medium supplemented with specific growth factors. As a result of asymmetric stem cell division spheroids were enriched in cancer stem cells (CSC), but also held a percentage of differentiated cells approaching necrosis. It should be noted that CSC were able to undergo asymmetric division also *in vivo* because after injection in mice, they both produced differentiated cells that constituted the bulk of the tumor and self-renewed themselves as demonstrated by the presence of CD44^+^CD24^−^ cells in all excised tumors.

A striking difference in tumorigenic capacity was shown by the different incidence and latency of tumors originating from spheroid CSC or DU145 cells injection, with the former cell population being more tumorigenic. We suggest that the delay in tumor formation after the injection of 5,000 DU145 cells could be ascribed to the negligible number of CSC held in DU145 cells injected compared to the enriched amount of CSC contained in each spheroid. These findings and this suggestion were further confirmed by the injection of 3×10^6^ DU145 cells, which held a greater amount of CSC than 1 spheroid and generated tumors in just 1 week.

Importantly, the further *in vivo* evaluation of the biological behavior of CSC contained in spheroids revealed that the injection of spheroids in nude mice generated highly aggressive tumors that were able to infiltrate the adjacent tissues and showed high density of NE cells and substantial vascularization, both of which were established features associated with prostate tumor progression [6], [26]. Our findings are consistent with a previous report which showed by histological analysis that purified CD44^+^ PCa cells, which possess many features of CSC, generated more invasive and metastatic tumors than the corresponding CD44^−^ cells [25]. Interestingly, we observed that invasive tumors generated from spheroid injection showed low levels of E-cadherin and β-catenin, consistently with the prognostic value of these indicators, which inversely correlated with PCa invasiveness and survival [15], [27]. In confirmation of these findings, scarcely invasive tumors originating from the injection of 5,000 DU145 cells expressed high levels of E-cadherin and β-catenin, and the injection of 3×10^6^ DU145 cells generated non-invasive capsulated tumors expressing the highest levels of E-cadherin and β-catenin (data not shown). In addition, more invasive tumors generated from spheroids also showed higher levels of vimentin, which correlated with poorly differentiated and metastatic PCa [17]. These data provide novel interesting information about the characteristics of the cells held in tumors originated in mice from the injection of CSC and DU145 cells. In particular, it appeared evident that the phenotype of CSC and DU145 cells was different from that of the cells of the tumors that they generated which showed an opposite pattern of expression of E-cadherin, β-catenin and vimentin. To explain this apparent contradiction it is important to consider the broad plasticity of CSC whose differentiation capacity is influenced by environmental stimuli. With this in mind, of particular interest is the observation that tumors generated by the injection of spheroids were more aggressive than those produced from DU145 cells, suggesting that CSC had the potential to form very aggressive tumors only when they were injected deprived of differentiated cells. On the contrary, when CSC were injected together with differentiated cells (i.e., injection of bulk DU145 cells), their potential was not expressed, and the resulting tumors were less aggressive. We underline that the different levels of aggressiveness of tumors were independent of the number of CSC inoculated, as confirmed by the injection of 3×10^6^ DU145 cells, which contained the highest amount of CSC but generated non-invasive tumors. We rather believe that higher or lower levels of tumor aggressiveness depend on the absence or presence of DU145 cells together with CSC. Although obtained in a single model of prostate cancer, these findings provide important information indicating that CSC can be controlled by environmental mechanisms that induce them to differentiate into cells expressing a highly or scarcely aggressive phenotype, thus determining tumor aggressiveness. These results lead us to speculate whether conventional therapies aimed to decrease the tumor mass indiscriminately may facilitate tumor progression as a consequence of removal of the differentiated tumor cell population.

### Interactions between CSC and DU145 cells

**Figure 7:**
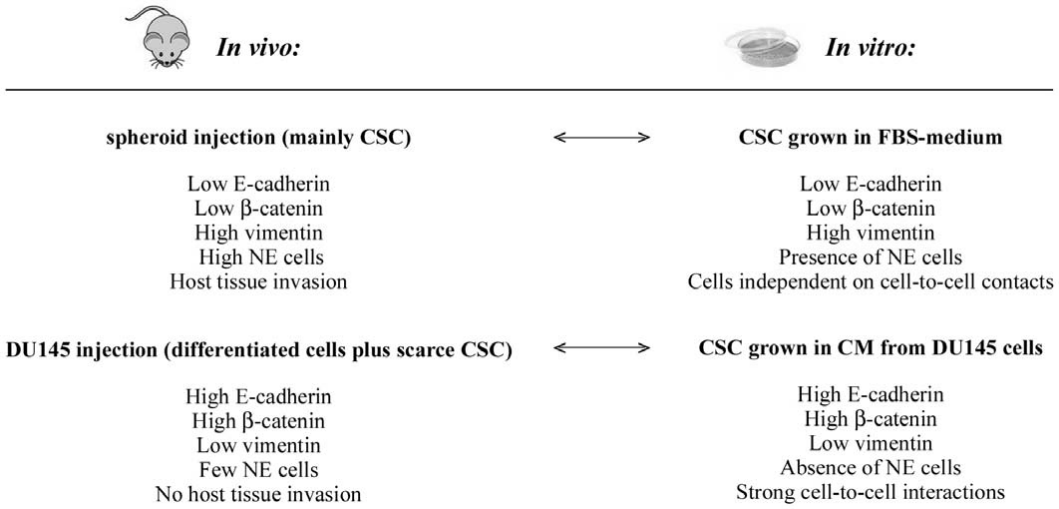
Parallelism between the phenotype of cells held in tumors and of CSC grown *in vitro*. In particular, CSC grown in FBS-medium aimed to reproduce the injection of 1 spheroid in mice, whereas CSC grown in CM from DU145 cells allowed us to simulate the injection of 5,000 unsorted DU145 cells.

In an attempt to explain the different features of tumors *in vivo*, we explored the pathways underlying the interactions between prostate CSC and differentiated DU145 cells *in vitro*. As pointed out in Figure 7, we observed a striking parallelism between the phenotypes of cells held in the 2 types of tumor generated in mice and those of the CSC grown in the 2 different culture conditions. In particular, molecular and cellular analyses demonstrated that CSC grown in permissive conditions *in vitro* underwent terminal differentiation producing cell populations with features similar to those of cells held in aggressive tumors generated from CSC injection in nude mice, namely NE cells and cells independent of cell-to-cell contacts, which were indistinguishable from DU145 cells. These findings further confirm the stem-like features of CD44^+^CD24^−^ cells analyzed in this study, since CSC differentiation was able to generate both prostate lineages, NE cells and epithelial cells. Moreover, it is interesting to note that because products released from NE cells enhanced the invasive potential of DU145 cells [28], NE cells could participate in the differentiation of CSC grown in FBS-medium towards a more aggressive phenotype. On the contrary, CSC grown in the presence of signals released from DU145 cells seemed to undergo an incomplete or alternative differentiation program resulting into a cell phenotype comparable to cells of scarcely aggressive tumors originated from the injection of bulk DU145 cells in mice. Therefore, our results provided evidence that the fate of prostate CSC was significantly context-dependent because the aggressive behavior of CSC was clearly controlled by cell-to-cell contacts as well as by soluble factors released from DU145 cells.

### Potentially relevant molecules in mediating the interactions between CSC and microenvironment

The importance of the interactions with the microenvironment to determine the phenotype of prostate CSC was confirmed by performing microarray and IPA analyses of spheroid CSC in comparison with total DU145 cells. In this way 22 genes particularly significant in the control of prostate CSC functions were selected most of which coded for products localized on the plasma membrane or secreted in the extracellular space. Many of these 22 genes have a well known role in PCa and were expressed in DU145 cells as expected in tumor cells. Interestingly, these genes were always inversely expressed in CSC and in DU145 cells. In particular, ANGPT2, CYR61 and VEGFC are involved in angiogenesis and are up-regulated in prostate cancer [29]–[31]. Other genes code for molecules involved in cell-to-cell adhesion, such as E-cadherin (CDH1), which is the main component of adherent junctions and whose reduced expression correlates with highly invasive PCa [11], [15]. JUP is a component of desmosomes and is less expressed in cancer with respect to normal prostate tissue [32]. ITGB6, which mediates the interactions between adjacent cells and between cells and extracellular matrix, is down-regulated during tumor progression [33]. CAV1 mediates the internalization of CDH1 and is up-regulated in prostate tumors [34]. Other genes act as tumor suppressors, such as IGFBP3, which inhibits prostate cancer growth through suppression of angiogenesis [35], and LCN2, which increases the expression of E-cadherin and suppresses cell invasiveness [36]. DPP4, KLF4 and TXNIP instead inhibit cell migration and invasion and induce cell cycle arrest [37]–[39]. Some of the selected genes play a relevant role in the maintenance of the stem cell niche. In particular, EGF induces stem cell differentiation, whereas BMP4 and TGFB2 induce quiescence [40]. These findings suggest that further investigations of the selected genes might allow to identify molecules that participate in determining the fate of CSC. In this regard, our preliminary results highlighted that the pathway of E-cadherin was clearly involved in the differentiation of prostate CSC. Notably, this last observation indicates that therapeutic manipulation of the adhesive interactions of prostate CSC could play an important role in the control of tumor progression.

Although some of the 22 selected genes have previously been identified in stem cells isolated in several different systems, only CDH1, KLF4 and ITGB6 have been correlated to the phenotype of prostate CSC [19], [23]. Therefore, in this study, we identified 19 potential novel markers associated with CD44^+^CD24^−^ prostate CSC that could be investigated for prospective new therapeutic applications after further evaluation in other experimental models.

### Conclusions

In the present study, we report for the first time that context-specific signals released from tumor microenvironment can induce CSC isolated from DU145 prostate carcinoma cell line to differentiate into mature tumor cells expressing a highly or scarcely aggressive phenotype, thus affecting prostate cancer progression. These findings encourage the identification of soluble factors present in the CM from DU145 cells that can be responsible for the control of the fate of the prostate CSC. For this purpose, the information gained by microarray analysis could be useful because it suggests a limited set of genes, most of which have never been considered before, potentially relevant in the interactions between CSC and the tumor microenvironment.

## Materials and Methods

### Ethics statement

Mice were purchased from Harlan Laboratories (Udine, Italy) and housed in the animal facility of the Regina Elena Cancer Institute, where there is currently no active ethical committee for animal research. Animals had *ad libitum* water and food and were maintained in standard conditions according to institutional guidelines under the control of the Italian Ministry of Health (DL 116/92).

### Cell cultures

DU145 cells (ATCC, HTB-81) were routinely cultured in DMEM medium (Invitrogen, Carlsbad, CA) supplemented with 10% FBS (HyClone, South Logan, UT) and 1% antibiotics (Sigma-Aldrich, Saint Louis, MO) in a humidified atmosphere at 37°C and 5% CO_2_.

### Sorting of CD44^+^CD24^−^ cells and cultures of spheroids

DU145 cells were incubated with anti CD44-PE and anti CD24-FITC antibodies (Invitrogen) on ice for 15 min, washed and resuspended in FACS buffer (PBS plus 1.5% BSA and 5 mM EDTA) and kept on ice until subsequent analysis on FACS Vantage-DIVA flow cytometer (excitation 488 nm, 100 mW, emission 530 nm BP (BrdU) and 610 nm LP (PI); Beckton Dickinson, San Jose, CA). Selected cells were seeded in 100 mm diameter plates at about 200,000 cells/dish in SRM consisting of DMEM/F12 medium (Invitrogen) supplemented with 20 ng/ml EGF, 10 ng/ml bFGF, 5 µg/ml insulin, 0.4% BSA and 1% antibiotics (Sigma-Aldrich). To avoid cell damage by centrifugations, 1 ml of fresh medium was added weekly in the plates until spheroids were generated. At that time, each spheroid was drawn with a tip and transferred to a new plate.

### Analysis of spheroid differentiation

Spheroids were mechanically fragmented and seeded in DMEM supplemented with 10% FBS. At different time points, cells were detached and counted using a Neubauer counting chamber. Photographs of the cells were taken under a phase contrast microscope (Leica Microsystems, Wetzlar, Germany).

### Western Blot analysis

To analyze protein expression in DU145 cells, control spheroids and spheroid cells grown in FBS-containing medium or CM, according to the experimental design, cell lysates were prepared by solubilizing cells in lysis buffer containing 20 mM Tris pH 8, 137 mM NaCl, 1% Nonidet P40, 10% glycerol, 10 mM EDTA pH 8 and a proteinase inhibitor cocktail (Complete TM, Roche Diagnostics GmbH Mannheim, Germany) for 20 min on ice. The supernatants were collected after centrifugation at 13,000 rpm for 15 min at 4°C. For protein extraction from mice tumors, frozen tissues were homogenized with an Ultra Turrax T 25 homogenizer in lysis buffer (2% SDS, 5 mM EDTA) supplemented with proteinase inhibitor cocktail. Homogenates were boiled for 10 min and clarified by centrifugation at 13,000 rpm for 15 min. The BCA Protein Assay Kit (Pierce, Rockford, IL) was used to measure protein concentration. Equal amounts of proteins (20 µg of cell lysates or 40 µg of tissue extracts) were subjected to 8% SDS-PAGE and electrophoretically transferred to PVDF membranes. Membranes were probed with monoclonal antibodies anti-E-cadherin (BD Transduction Laboratories, New Jersey, NJ), anti-β-catenin (BD), anti-vimentin (Santa Cruz Biotechnology, Santa Cruz, CA) and anti-β-actin (Sigma-Aldrich) followed by incubation in the presence of the appropriate secondary antibody conjugated to peroxidase (Bio-Rad, Hercules, CA). The signal was then detected by an enhanced chemiluminescence detection system.

### Transmission electron microscopy studies

Spheroids were fixed in 2.5% glutaraldehyde in phosphate buffer and then processed for TEM analysis following standard procedures [41].

### Evaluation of necrotic cells in spheroids

Spheroids were moved in a plate containing 1∶4 Trypan blue in SRM for 4 h, placed in PBS and photographed at phase contrast. Spheroid cells showing cytosolic blebs were spotted on a glass slide, stained with DAPI and analyzed by fluorescence microscope.

### Immunofluorescence

Ten spheroids were placed in a well of a vinyl multiwell plate (Costar, Cambridge, MA). The culture medium was carefully removed, and 100 µl of OCT compound (Tissue Tek, Sakura, Zoeterwoude, NL) was layered on spheroid sediments, which were snap frozen by contact with liquid nitrogen. After the removal of the vinyl coat, the embedded spheroids were cut (4 µm) in a Leica CM 1850 cryostat microtome. Following air drying, sections were fixed in absolute acetone for 5 min. The same fixing procedure was applied to 15,000 DU145 cells plated in an 8 well Tissue Chamber Slide (Nunc, Naperville, IL) and allowed to grow for 1 day. Primary antibodies and secondary fluoresceine-labeled antibodies that were employed for immunophenotyping were used at optimal dilutions established on positive and negative controls. Primary antibodies anti-E-cadherin, anti-β-catenin (BD Transduction Laboratories) and anti-vimentin (Santa Cruz Biotechnology) were used. Fluorescein-labeled goat anti rabbit IgG antibodies were purchased from Cappel Lab (Cochranville, PA), and fluorescein-labeled F(ab)2 rabbit anti mouse IgG were obtained from Sigma-Aldrich. Stained cells were analyzed employing a Leica DMIRE2 microscope equipped with a Leica DFC 350FX camera and elaborated by a Leica FW4000 deconvolution software (Leica, Solms, Germany).

Immunofluorescence staining for CD56 was performed on spheroid cells grown for 20 days in FBS-supplemented medium. Cells were fixed in 4% paraformaldehyde and permeabilized with 0.2% Nonidet P40. Primary antibody anti-CD56 (Epitomics, Burlingame, CA) and secondary fluoresceine-labeled antibody (Alexa Fluor 488, Invitrogen) were used. Nuclei were stained with Hoechst before analysis by fluorescence microscope.

### Mouse xenograft studies

Male NOD/SCID mice (6–8 weeks old, weight 22–25 g) were maintained for 2 weeks prior to each experiment. Before cell injection, animals were randomly allocated to one of the following groups: a) not injected, b) injected with 1 spheroid containing approximately 5,000 cells, c) injected with 5,000 unsorted DU145 cells, and d) injected with 3×10^6^ unsorted DU145 cells. Each group consisted of 10 animals. On ice, 125 µl DU145 cell suspensions or 125 µl SRM containing 1 intact spheroid were mixed with 75 µl matrigel. Mice were injected subcutaneously in the flank and monitored thrice every week for palpable tumor formation for a total of 100 days. Tumor size was measured with caliper and area was assessed by using the values of long axis and short axis. Once tumors reached an area between 1.2 and 1.5 cm^2^, mice were euthanized, and tumors were excised.

### Histological and immunohistochemical analysis

At sacrifice, a portion of tumor samples was fixed in 4% paraformaldehyde, paraffin-embedded and routinely stained by hematoxylin and eosin (H&E). CD56 immunohistochemical staining was carried out on 2 µm thick paraffin-embedded sections harvested on SuperFrost Plus slides (Menzel-Glaser, Braunschweig, Germany). The deparafinized and rehydrated sections were incubated with anti-CD56 primary antibody (Novocastra, Newcastle upon Tyne, UK) followed by anti-mouse biotinylated antibody and streptavidin-peroxidase complex (LSAB 2 system HRP, DakoCytomation, Milan, Italy) and 3,3′-diaminobenzidine (DAB substrate chromogen, DakoCytomation) was used as chromogen. To assess blood vessels density, 4 µm acetone-fixed cryostat sections were stained with anti-mouse CD31 monoclonal antibody kindly provided by Prof. A. Mantovani (Humanitas Inst. Milan, Italy) and MOM immunoenzymatic detection system purchased from Vectastain Labor (Burlingame, CA). Amino-ethyl carbazole was employed as chromogenic substrate. Nuclei were detected by slightly counterstain with Mayer hematoxylin and slides mounted in aqueous mounting medium (UCS Diagnostic, Rome, Italy). Vessels density was assessed by counting CD31 positive structures in at least 15 randomly selected microscopic fields at ×200 magnification. All morphological analyses were performed by two independent investigators.

### FACS analysis of tumors

Tumors excised from mice were collected in DMEM containing FBS and immediately processed. Tumors generated from spheroid injection needed an accurate removal of the adjacent murine tissues. Neoplastic tissues were dissociated mechanically and enzimatically using 2 mg/ml collagenase (Sigma-Aldrich) at 37°C for 90 min and sieved through 40 µm cell strainers. The cells were then resuspended in FACS buffer and incubated with anti CD44-PE and anti CD24-FITC antibodies for FACS analysis, as previously described.

### Conditioned medium experiments

DU145 cells were plated in 100 mm tissue culture dishes in 10 ml DMEM medium supplemented with FBS and grown for 3 days until subconfluence. The third day cells were harvested and plated again at the starting density in fresh medium for another 3 days, whereas the medium removed from the plates was used as CM from DU145 cells after being centrifuged at 1,000 rpm for 5 min and filtered through a 0.2 µm filter. This protocol was repeated for the duration of the experiment (20 days). In parallel, 4 spheroids were fragmented and cultured in CM or in DMEM containing FBS. The 2 culture media were replaced every 3 days with fresh CM or FBS-supplemented medium up to 20 days. To increase the concentration of soluble factors released from DU145 cells in CM a double number of DU145 cells were cultured in 10 ml FBS-containing medium for 3 days. As previously described, every 3 days cells were harvested and plated again at the starting density, whereas the collected medium was used as enriched CM to grow CSC contained in spheroids for a total of 20 days. On days 5, 10, 15 and 20, the proliferation of cells was measured with a Neubauer counting chamber. The cell culture morphology was observed under an optical microscope.

### RNA extraction

Total RNA was extracted from DU145 cells, control spheroids (groups of at least 30 spheroids of 400 µm in diameter or smaller) and spheroid cells grown in FBS-containing medium or in CM, according to the experimental design, through RNeasy Plus Micro Kit (Qiagen, Valencia, CA). For RNA extraction from mice tumors, frozen tissues were homogenized with an Ultra Turrax T 25 homogenizer in Trizol reagent (Invitrogen) and processed according to the manufacturer's instructions. RNA quantity and purity were assessed using a NanoDrop® instrument (Thermo Fisher Scientific Inc., Wilmington, DE). Total RNA integrity was assessed by Experion RNA Standard Sense kit (BioRad), and the RNA Integrity Number was calculated.

### Microarray hybridization and data analysis

Each condition was replicated 3 times. After extraction from spheroids and DU145 cells and quality checks, 70 ng of total RNA was retrotranscribed to cDNA by using the Applause™ WT-Amp Plus ST RNA Amplification Systems (NuGEN Technologies, San Carlos, CA). Five microliters of cDNA was fragmented and biotin-labeled by the Encore™ Biotin Module (NuGEN Technologies) following the procedure described by the manufacturer. The resulting biotin-labeled library was hybridized on GeneChip® Exon 1.0 ST human microarrays (Affymetrix, Santa Clara, CA). The CEL files resulting from the hybridization were analyzed using oneChannelGUI 1.6.5 [42]. Gene-level calculations were performed by Robust Multichip Average [43] and normalization by quantile sketch [44]. To assess differential expression at the gene-level, we used an empirical Bayes method [45] together with a false discovery rate (FDR) correction of the p-value [46]. Thus, the list of differentially expressed genes was generated using an FDR≤0.05 together with an absolute log_2_-(fold-change) threshold of 1. Differential expression was detected by linear model statistics. This method is based on the fitting of a linear model to estimate the variability in the data. In the case of one-channel microarray data, this approach is the same as the analysis of variance except that a model is fitted for every gene. For the detection of the differential expression, an empirical Bayes method is used to moderate the standard errors. The use of moderated statistics for the detection of differential expression is especially useful in cases of experiments with a small number of replicates.

The data discussed in this publication have been deposited in the NCBI Gene Expression Omnibus [47] and are accessible through GEO Series accession number GSE28313.

### cDNA synthesis and quantitative PCR

RNA was reverse-transcribed to single strand cDNA using random primers and Superscript II (Invitrogen). The ABI Prism 7000 Sequence Detection System and all reagents for Q-PCR were purchased from Applied Biosystems (Foster City, CA). Primers and probes for human CD44, CD24, CGA, CDH1, CYR61, DPP4, IGFBP3, ITGB6, LCN2, TGFB2 and mouse CD31, and for the endogenous reference genes human glyceraldehyde-3-phosphate dehydrogenase (GAPDH) and mouse β2-microglobulin (B2M) were purchased as TaqMan Gene Expression Assays. cDNA template was amplified in a reaction mixture containing TaqMan Universal PCR master mix and primers/probe mixture according to the manufacturer's default cycling conditions. For each amplification reaction, a standard curve was generated using serial dilutions (200, 40, 8, 1.6, 0.32 ng) of reverse-transcribed RNA extracted from DU145 cells or tumors generated from spheroid injection, according to the experimental design, and run concurrently with the test samples. All amplification reactions were performed in triplicate. Target gene expression was normalized to GAPDH or to B2M to correct for differences in the quantity of cDNA used for the amplification reactions.

### Statistical analysis

Mean values ± SEM from 3 independent experiments are shown. Statistical analysis was performed using the unpaired Student's *t*-test. *P* values of <0.05 were considered significant.

## Supporting Information

Table S1 **List of differentially expressed genes in CSC and DU145 cells obtained by microarray analysis.** Fold change represent the differential expression level of each gene in spheroid CSC with respect to DU145 cells. Genes listed in red are over-expressed in CSC, whereas genes showed in green are less expressed.
(XLS)
